# Supplementary material for: Identification and functional analysis of non-coding regulatory small RNA FenSr3 in Bacillus amyloliquefaciens LPB-18
Source: PeerJ. 2023 May 15;11:e15236. doi: 10.7717/peerj.15236 (PMC10194069; doi:10.7717/peerj.15236)
Supplement: Supplemental Information 4 [file peerj-11-15236-s004.zip › KO/CK-vs-T1_map/map04530.html]

KEGG PATHWAY: Tight junction - Reference pathway


|  |  |
| --- | --- |
| **Tight junction - Reference pathway** |  |

[
Pathway menu
| Organism menu
| Pathway entry
| Show description
| User data mapping
]

Tight junctions (TJs) are essential for establishing a selectively permeable barrier to diffusion through the paracellular space between neighboring cells. TJs are composed of at least three types of transmembrane protein -occludin, claudin and junctional adhesion molecules (JAMs)- and a cytoplasmic 'plaque' consisting of many different proteins that form large complexes. These are proposed to be involved in junction assembly, barrier regulation, cell polarity, gene transcription, and other pathways.

|  |  |  |
| --- | --- | --- |
| Reference pathway | 184% 150% 122% 100% 82% 67% 55% | 图片下载 |
